# Elevated Expression of Matrix Metalloproteinase-9 not Matrix Metalloproteinase-2 Contributes to Progression of Extracranial Arteriovenous Malformation

**DOI:** 10.1038/srep24378

**Published:** 2016-04-14

**Authors:** Ting Wei, Haihong Zhang, Neslihan Cetin, Emily Miller, Teri Moak, James Y. Suen, Gresham T. Richter

**Affiliations:** 1 Center for Investigation of Congenital Anomalies of Vascular Development, Arkansas Vascular Biology Program, Arkansas Children’s Hospital, University of Arkansas for Medical Sciences, Little Rock, AR, USA; 2 Department of Pathology, Arkansas Children’s Hospital, University of Arkansas for Medical Sciences, Little Rock, AR, USA; 3 University of Arkansas, Fayetteville, AR, USA; 4 University of Arkansas for Medical Sciences, Little Rock, AR, USA; 5 Department of Otolaryngology-Head and Neck Surgery, University of Arkansas for Medical Sciences, Little Rock, AR, USA; 6 Division of Pediatric Otolaryngology, Arkansas Children’s Hospital, Little Rock, AR, USA

## Abstract

Extracranial arteriovenous malformations (AVMs) are rare but dangerous congenital lesions arising from direct arterial-venous shunts without intervening capillaries. Progressive infiltration, expansion, and soft tissue destruction lead to bleeding, pain, debilitation and disfigurement. The pathophysiology of AVMs is not well understood. Matrix Metalloproteinases (MMPs) are thought to play an important role in pathologic processes underlying many diseases. This study investigates the expression of MMP-9 and MMP-2 in aggressive extracranial AVMs. The differential expression of MMP-9 and its regulatory factors is also examined. Herein we demonstrate that mRNA and protein expressions of MMP-9, but not MMP-2, are significantly higher in AVM tissues compared to normal tissues. The serum level of MMP-9, but not MMP-2, is also elevated in AVM patients compared to healthy controls. MMP-9/neutrophil gelatinase-associated lipocalin (NGAL) complex is also significantly increased in AVM tissues. The MMP-9/ tissue inhibitor of metalloproteases-1 (TIMP-1) complex presents as a major form detected in normal tissues. The increased and aberrant expression of MMP-9 and specific MMP-9 forms may help explain the constitutive vascular remodeling and infiltrative nature of these lesions. Specific MMP-9 inhibitors would be a promising treatment for AVMs.

Extracranial arteriovenous malformations (AVM) are congenital lesions thought to arise from the early and inappropriate communication between arteries and veins without an intervening capillary system. This rare but devastating condition may occur anywhere in the body with the head and neck being the most commonly affected sites. AVMs are present at birth and expand by continuous and infiltrative growth across normal anatomic boundaries to involve the skin, subcutaneous tissue, muscle and bones. Some lesions may be asymptomatic until later in life when environmental triggers stimulate their rapid and relentless enlargement. AVMs cause pain, sporadic and diffuse bleeding, disfigurement, and functional deficits in both children and adults. Life threatening bleeding and heart failure are the end results of their chronic growth. Unfortunately, treatment options for AVMs are limited and unsatisfactory with reported recurrence rates above 80% with surgical or intravascular therapies^1^,^2^. Pharmaceutical options are currently unavailable due to our limited understanding of the etiology and pathophysiology of this rare disorder.

Matrix metalloproteinases (MMPs) comprise a family of membrane bound and extracellular zinc-dependent endopeptidases involved in matrix degradation and tissue remodeling, particularly around normally and abnormally developing vasculature. MMPs are also involved in the activation and processing of factors relevant to angiogenesis and vascular stability, including TNF-α, TGF-β and VEGF^3^. Two members of the MMP family, MMP-9 and MMP-2, have been specifically discovered to have a profound impact on angiogenesis and vascular remodeling in malignancies, wound healing, vascular lesions, cardiovascular diseases and inflammatory disorders^4^,^5^,^6^,^7^,^8^. Their activities are tightly regulated by the enzyme stablizer-neutrophil gelatinase-associated lipocalin (NGAL) and inhibitor-tissue inhibitor of metalloproteases-1 (TIMP-1)^9^,^10^,^11^,^12^.

The aim of the current study was to investigate the role of MMP-9 and MMP-2, as well as their regulatory proteins, in actively expanding extracranial AVMs. Using combined molecular technologies, we discovered that the mRNA and protein expressions of MMP-9, but not MMP-2, were abnormally elevated in human extracranial AVMs. Four MMP-9 forms were discovered in AVMs including MMP-9 monomer, MMP-9 multimer, MMP-9/NGAL complex and MMP-9/TIMP-1 complex. The first three forms showed elevated expression in AVMs compared with normal samples. MMP-9/TIMP-1 complex presented as a major form detected in normal tissues. The serum level of MMP-9, but not MMP-2, was also increased in AVM patients compared to healthy matched controls.

## Results

### Messenger RNA expression of MMP-9, MMP-2, NGAL and TIMP-1

**Figure 1:**
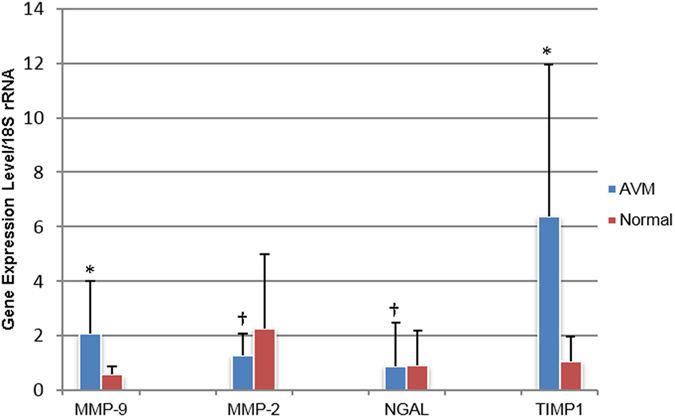
Messenger RNA expression profile. Comparison of mRNA expression levels of MMP-9, MMP-2, NGAL and TIMP-1 between AVMs and normal tissues. *significant difference; ^†^no significant difference; M/T, MMP-9/TIMP-1 complex; M/N, MMP-9/NGAL complex.

Messenger RNA expression levels of MMP-9, MMP-2, NGAL and TIMP-1 are presented in Table 1 as Mean ± SD and illustrated in Fig. 1. The range of expression value and median are also presented. The individual mRNA data were presented in Table 2. The amplification amount of all target genes was normalized against that of eukaryotic 18 S rRNA.

### Protein expression of MMP-9, MMP-2, NGAL and TIMP-1

**Figure 2:**
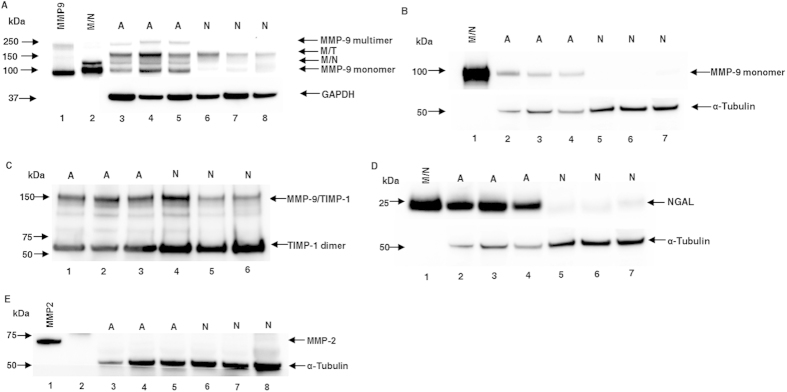
Western blot analysis of MMP-9, TIMP-1, NGAL and MMP-2 in AVMs and normal tissues. (**A**) Under non-reducing conditions, rhMMP-9 (lane 1) and purified neutrophil MMP-9/NGAL complex (lane 2) were used as positive controls; lanes 3–5, AVMs; lanes 6–8, normal tissues. (**B**) Under reducing conditions, purified neutrophil MMP-9/NGAL complex (lane 1) was used as a positive control (MMP-9/NGAL complex will break into MMP-9 and NGAL when running under reducing condition); lanes 2–4, AVMs; lanes 5–7, normal tissues. (**C**) Under non-reducing conditions, the blot was probed with anti-TIMP-1 antibody, lanes 1–3, AVMs; lanes 4–6, normal tissues. (**D**) Under reducing conditions, purified neutrophil MMP-9/NGAL complex was loaded as a positive control (lane 1); lanes 2–4, AVMs; lanes 5–7, normal tissues. (**E**) Under reducing conditions, rhMMP-2 was loaded as a positive control (lane 1); lanes 3–5, AVMs; lanes 6–8, normal tissues. A, AVM; N, normal tissue; M/T, MMP-9/TIMP-1 complex; M/N, MMP-9/NGAL complex.

**Figure 3:**
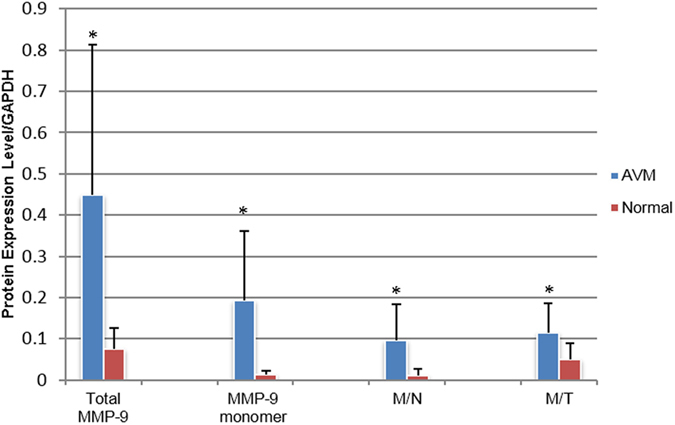
Protein expression profile. Comparison of protein expression levels of MMP-9 and its complexes between AVMs versus normal tissues. *significant difference; M/T, MMP-9/TIMP-1 complex; M/N, MMP-9/NGAL complex.

Under non-reducing conditions, blots with anti-MMP-9 antibody revealed four MMP-9 forms with molecular weights of ~250 kDa, ~150 kDa, ~125 kDa and ~92 kDa reproducibly detected in all of our AVM samples. These molecular weights corresponded with MMP-9 multimer, MMP-9/TIMP-1 complex, MMP-9/NGAL complex and MMP-9 monomer, respectively, based on the comigration positions of rhMMP-9 and purified neutrophil MMP-9/NGAL complex. In normal tissues, there was a significantly lower level of MMP-9 expression. The major form discovered in normal samples was MMP-9/TIMP-1 complex. The MMP-9 multimer was not detected in any of the normal tissues, which showed weak expression of the MMP-9/NGAL complex and MMP-9 monomer (Fig. 2A). Quantitative analysis showed that the expression levels of total MMP-9, MMP-9/TIMP-1 complex, MMP-9/NGAL complex and MMP-9 monomer in AVM tissues were statistically higher than those of the normal tissues using GAPDH as a loading control (Fig. 3). These data were presented in Table 3 and Table 4. The proportion of tissue samples showing positive expression of MMP-9 multimer was significantly higher in AVMs compared with normal tissues (10/11 vs. 0/10, *P* = 0.000).

Under reducing conditions, only the ~92 kDa MMP-9 form was detected in AVM tissues, and its expression was significantly higher than that of normal tissues using α-tubulin as a loading control (0.281 ± 0.034 vs. 0.005 ± 0.002, *P* = *0.03*) (Fig. 2B).

Under non-reducing conditions, blots with anti-TIMP-1 antibodies illustrated two bands at ~150 kDa and ~60 kDa corresponding to MMP-9/TIMP-1 complex (MMP-9 binding with TIMP-1 dimer) and TIMP-1 dimer, respectively, in both AVM and normal tissue groups (Fig. 2C). Under reducing conditions, higher expression at ~23 kDa corresponding to NGAL was detected in AVMs, which was statistically higher than that of normal tissues using α-tubulin as a loading control (5.706 ± 1.925 vs. 0.161 ± 0.026, *P* = *0.038*) (Fig. 2D). Blots with anti-MMP-2 antibodies revealed that no MMP-2 expression was detected in either AVMs or normal tissues (Fig. 2E).

### Gel Zymography

**Figure 4:**
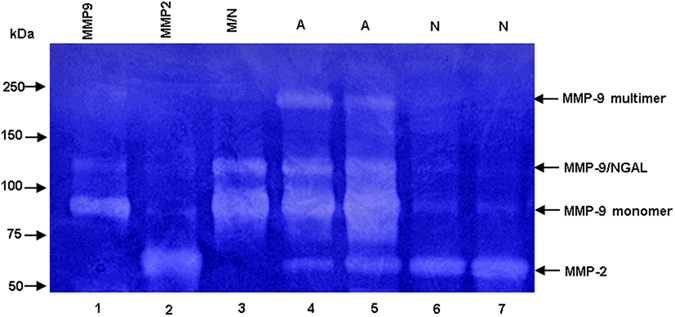
Gel zymography analysis of enzymatic levels of MMP-9 and MMP-2 in AVMs and normal tissues. RhMMP-9 (lane 1), rhMMP-2 (lane 2) and purified neutrophil MMP-9/NGAL complex (lane 3) were used as positive controls; lanes 4–5, AVMs; lanes 6–7, normal tissues.

**Figure 5:**
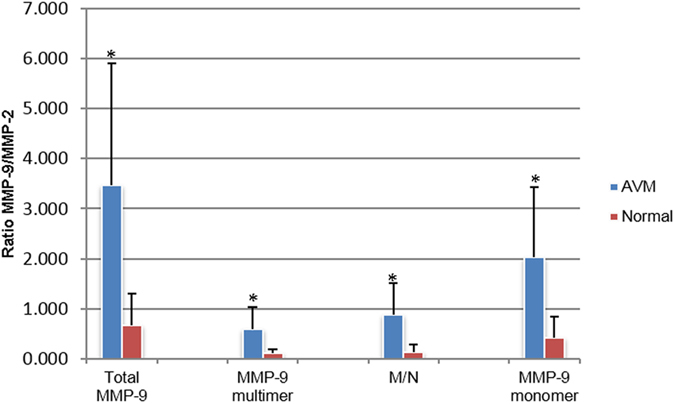
Comparison of enzymatic levels of MMP-9 in AVMs and normal tissues. Comparison of enzyme levels of MMP-9 and its complex normalized to enzyme level of MMP-2 between AVMs versus normal tissues. *significant difference. A, AVM; N, normal tissue; M/N, MMP-9/NGAL complex.

In gel zymography, clear bands against dark blue background indicate the gelatinase levels including proform and active form^13^. Four major clear bands were readily detected in all of the AVM samples, with molecular weights of ~250 kDa, ~125 kDa, ~86 kDa and ~68 kDa (Fig. 4). According to the comigration positions of rhMMP-9, rhMMP-2 and purified MMP-9/NGAL complex, the ~250 kDa and ~86 kDa bands were consistent with the MMP-9 multimer and monomer, respectively. The ~125 kDa bands corresponded to MMP-9/NGAL complex. The ~68 kDa bands represented MMP-2, which was detected in all AVM and normal samples with similar intensity reflective of normal endogenous MMP-2 expression (7.010 ± 5.582 in AVMs vs. 7.301 ± 4.272 in normal tissues, *P* = 0.96). This constitutive presence of MMP-2 allowed us to compare MMP-9 levels between AVMs and normal tissues and to express these levels as a ratio between MMP-9 and MMP-2^14^ (Table 5, Table 6 and Fig. 5). Statistical differences were found between AVMs and normal tissues with regard to the different MMP-9 forms.

### Immunohistochemistry

**Figure 6:**
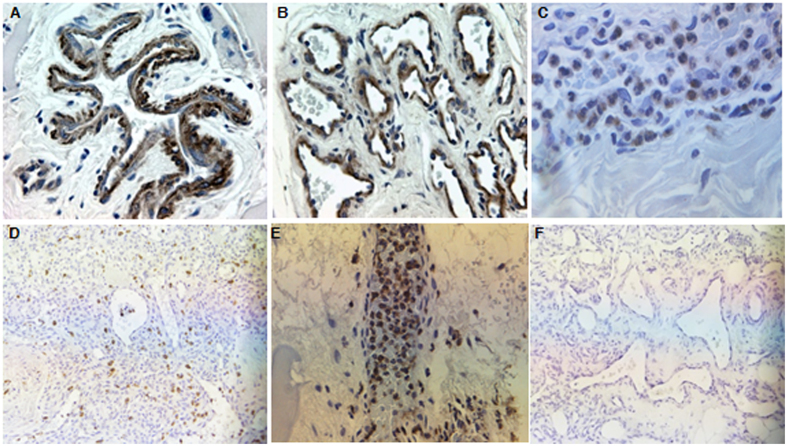
Immunohistochemistry analysis of MMP-9, NGAL and MMP-2 in AVMs. (**A–C**), MMP-9 was predominantly located in perivascular cells, endothelial cells and neutrophils. (**D**,**E**), abundant NGAL positive neutrophils were widely scattered in AVM tissues. (**F**), MMP-2 staining was negative in all AVMs and normal tissues. Original magnification ×400 (**A**–**C**) and ×200 (**D**–**F**).

MMP-9 staining was positive in the majority of AVM samples (9/11) compared with normal tissues (0/10), and was predominantly located in perivascular cells, endothelial cells and neutrophils (Fig. 6A–C). The staining indices based on staining intensity × staining percentage were 5.333 ± 4.387 (0 ~ 9, median 9) in AVMs vs.0.000 ± 0.000 (0 ~ 0, median 0) in normal tissues (*P* = 0.007). Abundant NGAL positive neutrophils were widely scattered in AVM tissues (Fig. 6D,E). MMP-2 staining was negative in all AVMs and normal tissues (Fig. 6F).

### Enzyme-linked Immunosorbent Assay

**Figure 7:**
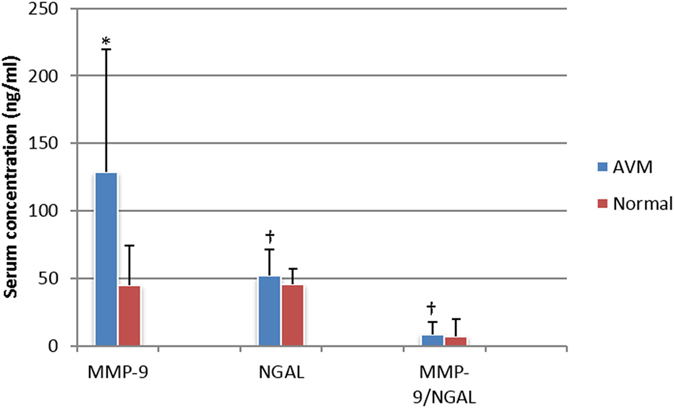
ELISA assay of serum concentrations of MMP-9, NGAL and MMP-9/NGAL complex in AVM patients versus normal controls. *significant difference; ^†^no significant difference.

**Figure 8:**
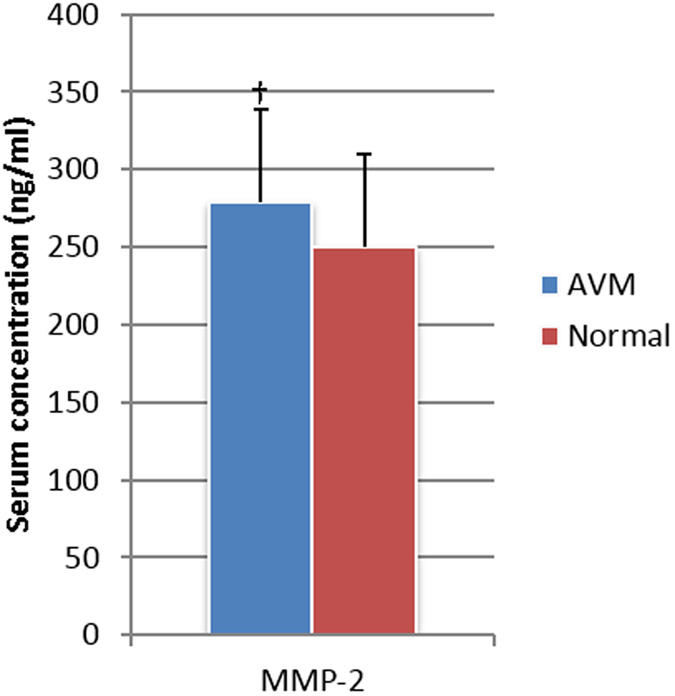
ELISA assay of serum concentrations of MMP-2 in AVM patients versus normal controls. ^†^no significant difference.

The serum levels of MMP-9 in AVM patients were significantly higher than those of healthy normal controls (*P* = 0.005), whereas the serum levels of MMP-2, NGAL and MMP9/NGAL complex in AVM patients were not significantly different from those of healthy controls (*P* = 0.14, *P* = 0.37 and *P* = 0.68, respectively, Figs 7 and 8, Tables 7 and 8.

## Discussion

Extracranial AVMs are thought to arise from errors in vascular morphogenesis. Histologically, they are composed of numerous aberrant arteriovenous shunts. Vascular recruitment and collateralization are thought to contribute to their progressive expansion, which makes active matrix remodeling as an important underlying mechanism. MMPs comprise a family of zinc-dependent endopeptidases involved in the matrix degradation and tissue and vascular remodeling. Two members of this family in particular, MMP-9 and MMP-2, are involved in diverse biologic processes and tightly regulated by gene expression, protein secretion, zymogen activation, and protein stabilization and inhibition. Loss of this regulation leads to a dysregulated process of matrix degradation or accumulation.

Herein we described the roles of MMP-9 and MMP-2 in the pathologic process of AVMs. We suspected involvement of these two MMPs due to the vast amount of histologically dysregulated and destabilized vascular elements and surrounding soft tissue found in AVM samples during any phase of their development or clinical presentation. To our knowledge, this is the first study to explore the relationship between MMPs and the pathophysiology of extracranial AVMs. We discovered that mRNA and protein expressions of MMP-9 are abnormally elevated in human extracranial AVMs. Similarly, circulating MMP-9 levels are elevated in AVM patients. The production of MMP-9 arises from aberrant vascular endothelial cells, perivascular cells and infiltrating neutrophils as demonstrated by the diffuse and high levels of expression seen in and also quantified by immunohistochemistry in these cell types from extracranial AVM samples compared with normal tissues. These cell sources of MMP-9 collude with the presumed pathogenesis of AVMs as a process of abnormal vascular remodeling and perivascular inflammation^15^.

We found that neither mRNA nor protein expression of MMP-2 differed between the AVMs and normal tissues. In fact, no MMP-2 protein signal is detected by Western blot or Immunohistochemistry assays in AVMs or normal samples. Physiologic levels are detected by gel zymography without significant difference between the AVM and normal tissue groups. Thus, MMP-2 is unlikely a causative factor in the pathogenesis of AVMs.

In human tissues and biological fluids, MMP-9 can be found as a monomer, a multimer, complexes with other molecules and as a low-molecular weight truncated form. In this study, we detected all of the MMP-9 forms in our samples except the truncated form. There was some debate about the identity of the multimers^16^. Vandooren and colleagues demonstrated the nature of the homomultimer is a reduction-sensitive trimer^17^. The authors also demonstrated that TIMP-1 free trimer has the same efficiency to induce angiogenesis as its monomer, but is more sensitive to TIMP-1 inhibition than its monomer. This MMP9 trimer could theoretically serve as a quick TIMP-1 trap, allowing the less sensitive MMP-9 monomer to induce angiogenesis^17^. We have conservatively elected to call this MMP-9 form a multimer in the present paper.

MMP-9 is heavily involved in tissue remodeling through the degradation of extracellular matrix substrates like collagen type IV and elastin^18^,^19^. MMP-9 has several other functions: it can convert cytokines and chemokines into active or inactive forms; process membrane-bound and intracellular proteins like intercellular adhesion molecule-1 (ICAM-1), occludins, actin and tubulin^20^,^21^,^22^,^23^; and release VEGF-A, a proangiogenic factor, from extracellular matrix^24^. As a result, MMP-9 is instrumental during normal physiologic conditions, such as embryo implantation, wound healing, vascular development, and bone growth^16^. The abnormal expression and activity of MMP-9, and/or a loss of the balance between MMP-9 and its endogenous regulators such as NGAL and TIMP-1, can disrupt normal arterial development and reorganization. Thus MMP-9 has been implicated in a host of human arterial disorders. For example, abnormalities in MMP-9 expression contribute to atherosclerotic plaque rupture^25^ and development of abdominal aortic aneurysms (AAA)^11^. AAA degeneration may be prevented by MMP inhibition or the overexpression of TIMP-1^26^. The present study suggests that AVM expansion is also linked to high levels of MMP-9 in its monomer and multimer forms and when stabilized by the NGAL protein.

NGAL is a ~25 kDa glycoprotein that belongs to the lipocalin super-family and is predominantly expressed by neutrophils. NGAL is thought to have a crucial role in vascular remodeling and influence the activity of MMP-9 through disulfide binding to form a ~125 kDa MMP-9/NGAL complex^10^,^27^. The MMP-9/NGAL complex is a typical product of neutrophils, which are a unique cell type that do not synthesize MMP-2 or TIMP-1, giving them a high angiogenic capacity^28^,^29^. MMP-9/NGAL complex expression and activities have been detected in tissue and urine samples from patients with vascular anomalies and cancers^6^,^30^,^31^,^32^,^33^. The presence of this complex has been associated with more advanced clinical stages of cancer and considered to play a key role in tumor invasion and metastasis as an independent predictor for tumor prognosis^30^,^31^,^32^,^33^. The MMP-9/NGAL complex has also been detected in AAA tissues and thought to be related to their pathogenesis^11^. Recent *in vitro* evidence indicated that NGAL greatly attenuated extracellular degradation of MMP-9 and supported the presumed protective role of NGAL for MMP-9^34^. In this study, MMP-9/NGAL complex was detected in all of the AVM tissues with significantly higher levels when compared with normal tissues shown by Western blot and gel zymography assay, implicating that more durable MMP-9 activity via NGAL protection contributes to relentless infiltration of AVM lesions into adjacent tissue. We detected no difference between AVM patients and healthy controls when comparing circulating levels of MMP-9/NGAL complex, which implies that this specific form of MMP-9 exerts itself more locally and is linked with a disease state.

AVMs are a pathologic condition thought to be somewhat governed by inflammation and its mediators (cytokines, neutrophils, macrophages, etc), triggered by genetic/hemodynamic factors, and local hypoxia^15^,^35^. Histologically, abundant inflammatory cells can be found to infiltrate AVM tissue. In our study, histologic analysis showed abundant neutrophils scattered diffusely in AVM specimens (Fig. 5D). Moreover, VEGF-A is capable of inducing rapid recruitment of a subset of neutrophils from circulation which contain more MMP-9 than the ones recruited by inflammatory stimulators^14^. Messenger RNA expression of VEGF-A in AVM tissues was significantly increased in our study compared to the normal tissues (data not shown), which contributed to greater neutrophil infiltration and TIMP-1-free MMP-9 secretion. In addition to neutrophils, some subgroups of macrophages produce TIMP-1 deficient proMMP-9^36^. The synergy of the above factors makes them highly angiogenic.

TIMP-1 is an endogenous and specific inhibitor of MMP-9 that is able to halt the enzymatic activity of MMP-9. In most cell types, the expression of TIMP-1 is very low; latent MMP-9 usually binds to TIMP-1 before secretion except in a few specific cell types as mentioned above^37^. Under physiologic conditions, the expression levels of MMP-9 and TIMP-1 are well balanced, which leads to normal matrix degradation and accumulation. Under pathologic conditions, this balance is disturbed with resultant excessive matrix degradation and subsequent vascular growth^38^,^39^. AVMs are thought to be in a constitutive state of remodeling and growth despite their clinical manifestation, which may initially appear to be a state of slow expansion. Ultimately this imbalance leads to rapid growth and/or soft tissue destruction. In the present study, all patients had actively growing AVM. Gene expression analysis by quantitative RT-PCR demonstrated significantly elevated mRNA levels of both MMP-9 and TIMP-1 in extracranial AVMs compared with normal tissue samples. Even though the mRNA level of TIMP-1 was increased in AVMs, the overexpression of MMP-9 likely was able to bypass the subsequent TIMP-1 overexpression, leading to the remodeling imbalance, excessive matrix degradation and vascular remodeling.

In this study, gel zymography showed three MMP-9 forms (MMP-9 multimer, MMP-9/NGAL complex and MMP-9 monomer) to be elevated in AVM samples compared with normal tissue samples. Because MMP-9/TIMP1 complex is dissociated by the presence of SDS during the eletrophoresis^13^, no clear bands were found at their expected positions. In contrast to Western blot assay, MMP-2 levels were detected in all samples with no significant difference between the AVMs and normal tissues. These findings suggest that a normal physiologic level of MMP-2 related to tissue remodeling is present under normal conditions. Because gel zymography detects inactive as well as active forms of the proteins tested, it should be noted that the results in the present study demonstrate the enzyme levels and not necessarily enzymatic activities of the MMP-9 forms and its complexes^13^.

The weakness of this study is the small sample size secondary to the rarity of AVM cases, which is probably less than 1:100,000 individuals. However, all samples came from pathologically and radiographically confirmed, actively growing AVMs requiring intervention for control. As such, all could be grouped into a Schobinger classification III and all were removed, with or without preoperative embolization, with the same degree of manipulation and trauma. Therefore, we maintain the AVM samples can be treated as a whole. In addition, the MMP-9 serum levels were positively correlated to the tissue expression levels, with minimal standard deviation despite slightly different patient characteristics.

Since MMPs have been implicated in various pathophysiological processes, MMP inhibitors have been extensively investigated for treatment of these conditions. Unfortunately, these inhibitors have shown little clinical efficacy because of their low selectivity^16^. Our study was aimed at determining the specific MMP dysregulation in the pathogenesis of AVMs and providing clues to help select specific MMP inhibitors that may improve clinical efficacy and decrease side effects. Establishment of an appropriate animal model of AVM is our next step before carrying out pharmaceutic trials.

In conclusion, MMP-9, and not MMP2, is significantly increased in extracranial AVMs compared with normal tissues. MMP-9/NGAL complex is a specific form of MMP-9 that protects MMP-9 enzymatic activity. The abundance of MMP-9 expression and its specific active form are most likely an important mechanism of progressive and relentless growth of AVMs. We plan to elucidate further the upstream and downstream pathways of MMP-9 to identify additional possible targets that will be focal points for our future translational work to develop a therapy for refractory AVMs.

## Methods

### Specimens

Fourteen extracranial AVM patients were recruited into this study after their informed consents were obtained. Among the 14 patients, 11 received surgeries.3 out of the 11 patients had preop embolization. Their lesional tissues were collected. Ten normal skins with subcutaneous tissues were obtained for normal control group. No trauma or infection was discovered; bleeding was present in a few patients (5 out of 14) (Table 9). Clinical history and physical examination showed that all the lesions were in an actively growing state. A pathologist (N.C.) experienced with vascular anomalies further confirmed the diagnosis for each patient at the time of resection. Specimens were immediately frozen on dry ice and transferred to a −80 °C freezer for long-term storage or immersed into 10% neutral formalin for 24 hr followed by paraffin embedding. Sera were collected from 14 patients before embolization or resection and from 11 age- and gender- matched healthy volunteers as normal controls.

### Real-time RT-PCR

30 mg of tissue was used for RNA isolation by RNeasy Plus Kit (Qiagen). All RNA samples were then converted into cDNA by TaqMan Reverse Transcription Reagents Kit (Lifetechnologies) using random primers according to the manufacturer’s instruction. The real-time PCR amplifications were performed on ABI 7900HT System (Lifetechnologies). The thermal cycling conditions were 1 cycle of 50 °C for 2 min, 1 cycle of 95 °C for 10 min, followed by 40 cycles of 95 °C for 15 sec and 60 °C for 1 min. The total reaction volume of 10 μl contained 5 ng of cDNA template, 5 μl of 2 × PCR Master Mix (Lifetechnologies), and 0.5 μl of 20 × primers/probe of target genes or endogenous control assay mix. MMP-9 (Hs00234579_m1), MMP-2 (Hs01548727_m1), NGAL (Hs01008571_m1), and TIMP-1 (Hs00171558_m1) were purchased from Lifetechnologies. The Eukaryotic 18 S rRNA (Lifetechonolgies, Hs99999901_s1) was used as the endogenous control. The comparative Ct method was used to determine relative quantification. The amplification amount of all target genes was normalized against that of eukaryotic 18 S rRNA.

### Western blot analysis

Total proteins were extracted using T-PER tissue protein extraction reagent (Thermo Scientific) added with Halt Protease Inhibitor Cocktail (Thermo Scientific) and 1 mM EDTA (Invitrogen). Protein concentrations were measured using a BCA protein assay kit (Thermo Scientific). 1 ~ 30 μg of total protein was loaded on NuPAGE® Novex® 4–12% Bis-Tris Protein Gels (Invitrogen) for electrophoresis under non-reducing or reducing conditions and transferred to PVDF membranes (Bio-Rad). The membranes were blocked with 5% non-fat milk in TBST (Thermo Scientific) for 1 h at room temperature, followed by incubation with primary antibodies at 4 °C overnight. Blots were washed with TBST 3 times, followed by incubation with horseradish peroxidase conjugated secondary antibodies (Santa Cruz, 1:2000) for 1 h at room temperature. Blots were developed with Novex ECL Chemiluminescent Substrate Reagent Kit (Invitrogen) for 5 min in the dark and exposed for 2 min on Image Quant™ LAS 4000 (GE Healthcare). The primary antibodies used included mouse anti-human MMP-9 (Millipore, MAB13415, 1:200), goat anti-human MMP-9 (R&D, AF911, 1:500), mouse anti-human MMP-2 (R&D, MAB9022, 1:500), rabbit anti-human NGAL (Abcam, ab125075, 1:1000), rabbit anti-human TIMP-1 (Millipore, AB770, 1:200), rabbit anti-human GAPDH (Cell Signaling technology, 14C10, 1:1000) and rabbit anti-α-tubulin (Cell Signaling technology, 11H10, 1:1000). Recombinant human MMP-9 (R&D), MMP-2 (R&D), and purified human neutrophil MMP-9/NGAL complex (Millipore) were used as positive controls (under reducing conditions, MMP-9/NGAL complex can break down into MMP-9 and NGAL; NGAL can be used as a positive control when detecting NGAL expression in AVMs). Image results were analyzed with Image Quant TL 7.0 software (GE Healthcare).

### Gel Zymography

12 μg of total protein (same preparation as Western blot) was loaded on Novex® 10% Zymogram (Gelatin) Protein gels (Invitrogen) for electrophoresis. Gels were then renatured using Renaturing Buffer (Invitrogen) for 30 min at room temperature and equilibrated in Developing Buffer (Invitrogen) for 30 min at room temperature. After that, gels were incubated in Developing Buffer at 37 °C overnight. After a few brief rinses with distilled water, gels were stained with 0.1% Coomassie Blue (Sigma) in 40% methanol (Fisher Scientific) and 10% acetic acid (Fisher Scientific) for 30 min at room temperature and destained in 10% methanol and 7.5% acetic acid for 1–2 h at room temperature. Tap water was used to stop destaining. Gels were pictured on Image Quant™ LAS 4000 and results were analyzed with Image Quant TL 7.0 software. Recombinant human MMP-9, MMP-2 and purified neutrophil MMP-9/NGAL complex were used as positive controls.

### Immunohistochemistry

After deparaffinization and rehydration, sections were heated to 95 °C for 15 min in Citrate Buffer, pH 6.0 (Invitrogen) for antigen retrieval. 3% H_2_O_2_ (Fisher Scientific) was used to block endogenous peroxidase activity for 15 min. Sections were preincubated with 10% normal serum for 30 min at room temperature and, then incubated in primary antibodies at 4 °C overnight. Primary antibodies included rabbit anti-human MMP-9 (Cell Signaling technology, 3852, 1:200), mouse anti-human NGAL (Millipore, MABN481, 1:1000), and rabbit anti-human MMP-2 (Proteintech, 10373-2-AP, 1:50). After washing in PBST, sections were incubated in biotinylated secondary antibodies (Vector Labs, 1:200) for 30 min at room temperature followed by ABC reagent (Vector labs) for 30 min. Color was developed using ImmPACT™ DAB (Vector Labs). The sections were then counterstained with hematoxylin (Fisher Scientific), dehydrated, and mounted using permanent mounting media (Fisher Scientific). Slides with no primary antibody applied were used as the negative controls. The staining results were validated by a blind review performed by a pathologist (N.C.) with extensive experience examining vascular anomalies and Immunohistochemistry. Staining intensity was categorized into 4 levels: none (score 0), weak (score 1), moderate (score 2) and strong (score 3). Staining percentage of positive cells was categorized into 4 levels: <25% (score 0), 25–50% (score 1), 50–75% (score 2) and >75% (score 3). Staining index was calculated by multiplying staining intensity by staining percentage.

### Enzyme-linked Immunosorbent Assay

Peripheral whole blood samples were obtained from venipuncture and centrifuged at 1000 × g for 15 min after 30 min of clotting at room temperature. Serum was transferred, aliquoted, and stored at −80 °C until analysis. MMP-9, MMP-2, NGAL and MMP-9/NGAL complex serum levels were determined using commercially available ELISA kits according to the manufacturer’s instructions (MMP-9, NGAL and MMP-9/NGAL complex from R&D, MMP-2 from Invitrogen). Optical density was read on an iMark^TM^ microplate absorbance reader (Bio-Rad).

### Statistical analysis

The expression level was presented as the mean ± the standard deviation (SD), minimum value, maximum value and median. The significant difference between the AVM and normal tissue groups was calculated using the non-parametric Mann-Whitney U test when there was relatively wide range of expression levels among different individuals in both groups. Otherwise, the Student’s t-test was used when the data displayed normal distribution. Fisher’s exact test was used when comparing proportion differences between these two groups. The statistical analysis was run on an IBM SPSS Statistics 22. *P* < *0.05* was considered statistically significant.

### Study approval

This study was approved by the Institutional Review Board of the University of Arkansas for Medical Sciences, protocol number 114012. All experiments were performed in accordance with relevant guidelines and regulations. Written informed consent was received from the participants or their guardians prior to tissue and serum collection.

## Additional Information

**How to cite this article**: Wei, T. *et al.* Elevated Expression of Matrix Metalloproteinase-9 not Matrix Metalloproteinase-2 Contributes to Progression of Extracranial Arteriovenous Malformation. *Sci. Rep.* **6**, 24378; doi: 10.1038/srep24378 (2016).

## Figures and Tables

**Table 1:** mRNA expression levels of MMP-9, MMP-2, NGAL and TIMP-1 in AVMs and normal tissues.

Gene	Sample	Mean ± SD	Min Value	Max Value	Median	*P* Value
MMP-9	AVM	2.10 ± 1.93	0.03	5.78	1.60	*P* < 0.05
	Normal	0.58 ± 0.32	0.09	1.00	0.60	
MMP-2	AVM	1.28 ± 0.81	0.43	3.01	1.01	*P* > 0.05
	Normal	2.28 ± 2.72	0.52	8.83	1.00	
NGAL	AVM	0.87 ± 1.63	0.04	5.71	0.18	*P* > 0.05
	Normal	0.92 ± 1.29	0.03	3.77	0.48	
TIMP-1	AVM	6.39 ± 5.56	1.27	21.93	5.08	*P* < 0.05
	Normal	1.08 ± 0.91	0.29	3.30	0.76	

**Table 2:** Individual data of expression levels of MMP-9 mRNA in AVMs and normal tissues.

AVM	MMP-9 mRNA	Normal Tissue	MMP-9 mRNA
1	0.03	1	0.09
2	0.53	2	0.72
3	2.85	3	0.39
4	5.78	4	1.00
5	4.27	5	0.47
6	0.65	6	0.85
7	1.01	7	0.84
8	0.22	8	0.24
9	1.91	9	10.22*
10	4.23	10	13.03*
11	1.60		

^*^Note: Normal tissues 9 and 10 have abnormally high level of MMP-9 mRNA, histology examination shows lymphatic malformation invasion (9) and ear dermoid cyst (10). These two data were deleted when statistical analysis was conducted.

**Table 3:** Protein expression levels of MMP-9 and its complexes in AVMs and normal tissues.

Protein	sample	Mean ± SD	Min Value	Max Value	Median	*P* value
Total MMP-9	AVM	0.449 ± 0.363	0.170	0.446	0.381	*P* < 0.05
	Normal	0.075 ± 0.052	0.000	0.161	0.066	
MMP-9 monomer	AVM	0.194 ± 0.168	0.025	0.497	0.112	*P* < 0.05
	Normal	0.014 ± 0.008	0.000	0.024	0.013	
MMP-9/NGAL	AVM	0.095 ± 0.089	0.031	0.253	0.050	*P* < 0.05
	Normal	0.011 ± 0.016	0.000	0.048	0.005	
MMP-9/TIMP-1	AVM	0.114 ± 0.072	0.032	0.262	0.090	*P* < 0.05
	Normal	0.050 ± 0.039	0.000	0.095	0.046	

**Table 4:** Individual data of protein levels of MMP-9 and its complexes in AVMs and normal tissues.

AVM	Total MMP-9	MMP-9 monomer	MMP-9/NGAL	MMP-9/TIMP-1
1	0.216	0.078	0.049	0.069
2	0.320	0.070	0.073	0.177
3	0.446	0.172	0.093	0.139
4	1.245	0.470	0.284	0.161
5	0.412	0.290	0.050	0.044
6	0.134	0.025	0.031	0.075
7	0.381	0.112	0.094	0.164
8	0.171	0.092	0.031	0.032
9	1.039	0.497	0.253	0.262
10	0.170	0.043	0.033	0.090
11	0.406	0.286	0.050	0.041
Normal tissue				
1	0.115	0.021	0.000	0.095
2	0.084	0.015	0.000	0.069
3	0.000	0.000	0.000	0.000
4	0.161	0.024	0.048	0.089
5	0.048	0.012	0.014	0.023
6	0.042	0.011	0.012	0.019
7	0.110	0.020	0.000	0.090
8	0.039	0.010	0.010	0.019

**Table 5:** Enzyme levels of MMP-9 and its complexes in AVMs and normal tissues relative to MMP-2.

Enzyme	Sample	Mean ± SD	Min Value	Max Value	Median	*P* Value
Total MMP-9	AVM	3.461 ± 2.444	1.415	9.469	2.729	*P* < 0.05
	Normal	0.699 ± 0.633	0.030	1.491	0.322	
MMP-9 multimer	AVM	0.595 ± 0.451	0.194	1.797	0.479	*P* < 0.05
	Normal	0.109 ± 0.080	0.000	0.250	0.135	
MMP-9/NGAL	AVM	0.876 ± 0.631	0.090	2.334	0.680	*P* < 0.05
	Normal	0.135 ± 0.153	0.000	0.423	0.098	
MMP-9 monomer	AVM	2.030 ± 1.408	0.624	5.338	1.756	*P* < 0.05
	Normal	0.429 ± 0.411	0.030	1.021	0.184	

**Table 6:** Individual data of enzyme levels of MMP-9 and its complexes in AVMs and normal tissues.

AVM	Total MMP-9	MMP-9 multimer	MMP-9/NGAL	MMP-9 monomer
1	2.137	0.325	0.670	1.142
2	3.476	0.656	0.680	2.139
3	1.415	0.297	0.494	0.624
4	9.469	1.797	2.334	5.338
5	2.729	0.370	0.603	1.756
6	1.435	0.289	0.289	0.857
7	3.341	0.547	0.865	1.928
8	3.970	0.888	1.053	2.029
9	6.249	0.704	1.626	3.919
10	1.547	0.194	0.090	1.263
11	2.300	0.479	0.933	1.332
**Normal tissues**	**Total MMP-9**	**MMP-9 multimer**	**MMP-9/NGAL**	**MMP-9 monomer**
1	0.160	0.000	0.010	0.150
2	0.030	0.000	0.000	0.030
3	0.185	0.000	0.001	0.184
4	1.391	0.135	0.235	1.021
5	1.103	0.156	0.219	0.728
6	1.491	0.250	0.423	0.818
7	0.322	0.222	0.026	0.074
8	2.727*	1.156*	0.169	1.401*

^*^Note: normal tissue 8 has abnormally high enzyme level of MMP-9, its data were deleted when statistical analysis was conducted.

**Table 7:** Serum concentrations of MMP-9, NGAL, MMP-9/NGAL and MMP-2 (ng/ml).

Serum enzyme	Sample	Mean ± SD	Min Value	Max Value	Median	P Value
MMP-9	AVM	128.63 ± 90.80	3.15	377.77	123.19	*P* < 0.05
	Normal	44.79 ± 29.73	0.00	76.24	55.44	
NGAL	AVM	52.10 ± 19.12	20.74	91.70	46.51	*P* > 0.05
	Normal	45.47 ± 12.00	28.34	62.70	43.77	
MMP-9/NGAL	AVM	8.82 ± 9.07	0.00	30.84	6.26	*P* > 0.05
	Normal	6.77 ± 13.16	0.00	43.67	3.44	
MMP-2	AVM	279.00 ± 59.65	21.33	38.70	26.70	*P* > 0.05
	Normal	250.11 ± 59.76	15.87	34.39	24.18	

**Table 8:** Individual data of serum concentration of MMP-9 in AVMs and healthy normal controls (ng/ml).

AVM	Serum MMP-9	Healthy control	Serum MMP-9
1	3.149	1	73.761
2	144.754	2	65.173
3	152.006	3	76.242
4	377.771	4	46.661
5	221.281	5	29.867
6	43.989	6	22.615
7	142.845	7	2.577*
8	127.96	8	0
9	158.113	9	69.371
10	52.768	10	0
11	118.418	11	64.219
12	98.761		
13	70.516		
14	88.456		

^*^Note: Serum from normal control 7 showed obvious hemolysis, the result was deleted when statistical analysis was conducted.

**Table 9:** History of bleeding and Preop embolization of patients.

Patients	Bleeding	Surgery	Preop embolization
1	No	Yes	No
2	No	Yes	No
3	Yes	Yes	Yes
4	No	Yes	No
5	No	Yes	Yes
6	No	Yes	No
7	Yes	Yes	Yes
8	No	Yes	No
9	No	Yes	No
10	Yes	Yes	No
11	Yes	Yes	No
12	No	No	
13	No	No	
14	Yes	No	
